# Molecular control of non-muscle myosin II assembly

**DOI:** 10.18632/oncotarget.6936

**Published:** 2016-01-18

**Authors:** Alba Juanes-García, Clara Llorente-González, Miguel Vicente-Manzanares

**Affiliations:** Universidad Autonoma de Madrid School of Medicine and Instituto de Investigacion Sanitaria Hospital de Ia Princesa, Madrid, Spain

**Keywords:** myosin, actin, migration, polarity

Mechanical forces play fundamental roles in cellular processes related to movement. The major generator of mechanical forces in living systems is myosin II, which lies inside cells. Myosin II propels actin filament sliding, converting chemical energy into movement in various scales, from stress fibre formation and sliding to muscle contraction. In non-muscle cells, such forces are exerted at a cellular/subcellular scale and drive motility processes, e.g. cell migration, division, etc.

There are several isoforms of myosin II, divided into muscle, smooth muscle and non-muscle. They all consist of a hexamer made of two heavy chains (MHCII) and four light chains, two structural (ELC) and two regulatory (RLC). The heavy chain displays three domains: a globular, actin- and ATP-binding domain, a supercoiled long domain that mediates dimerization of the MHCII; and a non-helical tail domain. Non-muscle myosin II (NMII) appears as three isoforms, II-A, II-B and II-C depending on the MHCII. Their ability to generate mechanical forces is context-dependent, thereby each isoform regulates specific cellular functions. In addition, every cell type displays a specific pattern of expression that may evolve over time, e.g. during embryogenesis, being crucial for proper development.

Of the three isoforms, NMII-A is the fastest moving, although it can bear considerably less load than the other two. It displays fast assembly immediately behind the leading edge of migrating cells. Conversely, NMII-B and II-C are slower, but bear more load [1]. NMII-B assembles slowly in central, lateral and back regions of the cell and use pre-formed NMII-A-based filaments for their elongation. Selective depletion experiments have revealed specific cellular roles for these isoforms. NMII-A depletion impairs focal adhesion formation and retraction of the rear, whereas NMII-B depletion causes a marked loss of front-rear polarity in 2-D [2].

When not forming filaments, NMII is folded in a compact, assembly-incompetent conformation (10S) that extends by phosphorylation of the RLC on Ser19. The same phosphorylation triggers the conformational movement of the head of the heavy chain that enables actin filament movement [3]. Another level of regulation resides in the stability of the filaments that is controlled by the 10S-6S balance and the ability of the hexamers to form oligomers (mini-filaments) of 6-25 hexamers arrayed in an anti-parallel fashion [2].

A regulatory hotspot of the stability of the oligomers is the phosphorylation of the heavy chain. Unlike RLC phosphorylation, this is an isoform-specific mechanism. For example, the stability of the NMII-A filaments is controlled by phosphorylation of MHCII-A on S1943. Phospho-S1943 NMII-Amini-filaments are unstable, thus forming very small and weak filaments, and overall loss of cellular contraction [4]. Regarding NMII-B, S1935 is a novel phospho-regulatory residue that controls the dynamics of NMII-B filaments. When phosphorylated, NMII-B forms smaller mini-filaments that bear less load. In fact, assembled phospho-Ser1935 NMII-B behaves like assembled NMII-A, including its ability to nucleate mini-filaments de novo [5]. This motifis crucially important for the speed and load of the NMII isoforms, since its “transplantation” into the same spot in NMII-A endows it with NMII-B-like properties (larger filaments, higher load and decreased *de novo* mini-filament formation). S1935 is much closer to the domain-breaking Pro in NMII-B than S1943 is in NMII-A, suggesting different mechanisms that nevertheless have a similar outcome, i.e. decreased filament stability. In the case of NMII-A, S1943 phosphorylation regulates its interaction with S100A4/Mts1, which may control NMII-A assembly dynamics and stability [4]. We speculate that addition of a strong negative charge due to phosphorylation of S1935 next to the domain-breaking Pro (1933) compromises the stability of the bundled coiled-coil + non-helical domain pieces of NMII-B. As a consequence, filaments are less stably packed which may cause higher filament turnover disassembly. At a cellular level, a phosphomimetic mutation of S1935 impairs formation of the large actomyosin budles that define the backof the cell and a concomitant loss of directional polarity in 2-D, indicating that S1935 controls the formation of a stable directional rear that supports polarized migration [2].

The physiological implications of these mechanisms of regulation are just beginning to be elucidated. Phosphorylation of S1943 controls CD34+ hematopoietic stem cell fate [6]. Also, an inhibitory peptide from Met aminopeptidase-2 blocks the interaction of Mts1 with NMII-A, increasing NMII-A assembly and preventing tumor angiogenesis [7]. Whether S1935 phosphorylation directly controls the assembly of NMII-B or it does it by managing the interaction of NMII-B with accessory proteins remains to be shown. These studies suggest that the selective control of NMII assembly in an isoform-dependent manner is a central issue in processes involving symmetry breaking, motility and cell division.
